# Pulmonary Expression of Interleukin-17 Contributes to Neutrophil Infiltration into the Lungs during Pneumonic Plague

**DOI:** 10.1128/iai.00131-23

**Published:** 2023-06-20

**Authors:** Hayley M. Theriot, Priyangi A. Malaviarachchi, Madeleine G. Scott, Kenneth T. Appell, Srijon K. Banerjee, Roger D. Pechous

**Affiliations:** a Department of Microbiology and Immunology, University of Arkansas for Medical Sciences, Little Rock, Arkansas, USA; b Department of Microbiology and Molecular Genetics, University of Pittsburgh School of Medicine, Pittsburgh, Pennsylvania, USA; University of Pennsylvania

**Keywords:** IL-17, *Yersinia*, *Yersinia pestis*, plague, pneumonic plague

## Abstract

Inhalation of respiratory droplets infected with Yersinia pestis results in a rapidly progressing and lethal necrotic pneumonia called primary pneumonic plague. Disease manifests as biphasic, with an initial preinflammatory phase with rapid bacterial replication in the lungs absent readily detectable host immune responses. This is followed by the onset of a proinflammatory phase that sees the dramatic upregulation of proinflammatory cytokines and extensive neutrophil accumulation in the lungs. The plasminogen activator protease (Pla) is an essential virulence factor that is responsible for survival of Y. pestis in the lungs. Our lab recently showed that Pla functions as an adhesin that promotes binding to alveolar macrophages to facilitate translocation of effector proteins called Yops into the cytosol of target host cells via a type 3 secretion system (T3SS). Loss of Pla-mediated adherence disrupted the preinflammatory phase of disease and resulted in early neutrophil migration to the lungs. While it is established that *Yersinia* broadly suppresses host innate immune responses, it is not clear precisely which signals need to be inhibited to establish a preinflammatory stage of infection. Here, we show that early Pla-mediated suppression of Interleukin-17 (IL-17) expression in alveolar macrophages and pulmonary neutrophils limits neutrophil migration to the lungs and aids in establishing a preinflammatory phase of disease. In addition, IL-17 ultimately contributes to neutrophil migration to the airways that defines the later proinflammatory phase of infection. These results suggest that the pattern of IL-17 expression contributes to the progression of primary pneumonic plague.

## INTRODUCTION

Yersinia pestis is a Gram-negative bacterium responsible for bubonic, septicemic, and pneumonic plague (1). Primary pneumonic plague results from inhalation of infectious respiratory droplets and is 100% lethal if left untreated (1, 2). The progression of primary pneumonic plague is biphasic, with an initial preinflammatory phase marked by the lack of disease symptoms or significant host innate immune responses despite rapid bacterial replication in the lung (3–7). This is followed by the switch to a proinflammatory phase that sees the abrupt onset of host inflammatory responses that drive a severe and lethal necrotizing pneumonia (3–7). The two disease phases are largely defined by the presence or absence of proinflammatory cytokines/chemokines, neutrophils, and pulmonary pathology in the lungs. During the early preinflammatory phase Y. pestis suppresses proinflammatory cytokine production to limit neutrophil chemotaxis to the lungs. In contrast, progression into the proinflammatory phase of disease is defined by a proinflammatory cytokine storm and the massive and sustained influx of neutrophils to the airways that leads to pulmonary tissue damage, ultimately compromising pulmonary function and resulting in significant mortality (3, 8). While there is currently no licensed vaccine to prevent pneumonic plague, antibiotic treatment is successful when administered prior to and within 24 h of the onset of symptoms (9, 10). However, as a result of the rapid progression of pulmonary inflammation, antibiotic therapy delivered outside this time window has limited efficacy. Due to initial nondescript symptoms, pneumonic plague may go misdiagnosed, leading to high mortality rates.

Y. pestis has several virulence factors that contribute to pathogenesis during pneumonic plague (11). Among these is the plasminogen activator protease (Pla), which is required for bacterial survival in the lung (12–14). Pla is an outer membrane aspartate protease that cleaves several substrates, including plasminogen, Fas ligand, and plasminogen activation inhibitor 1 (15–17). Our lab recently showed that independent of its enzymatic function, Pla acts as an adhesin to facilitate targeting of alveolar macrophages for type three secretion (T3S)-mediated delivery of effector proteins termed *Yersinia* outer proteins (Yops) (13, 14). Upon injection, these effector proteins interfere with host production of proinflammatory cytokines and inhibit phagocytosis of the bacteria, allowing Y. pestis to replicate to high numbers in the lung absent appreciable innate immune responses or bacterial killing (11, 14, 18). Deletion of Pla resulted in the increased early infiltration of neutrophils into the lungs, thereby disrupting the initial preinflammatory phase of disease (13).

Interleukin-17A (IL-17A) is a proinflammatory cytokine that plays a role in protection against multiple pathogens (19, 20). Initially thought to be produced only by Th17 cells, IL-17 is produced by a number of adaptive and innate immune cells, including alveolar macrophages and neutrophils (19, 21, 22). In the context of extracellular bacterial and fungal infections, IL-17 protection is typically neutrophil-mediated (20, 21). Despite its role in protection against disease, dysregulation of IL-17 can also lead to excessive inflammation through the continuous recruitment of host innate immune populations (19, 23). During pulmonary infection with Y. pestis, IL-17 pathways are highly activated during later phases of disease and are thought to contribute to macrophage programming that aids in controlling bacterial survival (12, 24).

We sought to understand the bacterium-host cell dynamics that establish a preinflammatory phase of disease and, conversely, the abrupt switch to a proinflammatory phase. Specifically, we focused on identifying host molecules whose pattern of expression influenced neutrophil migration to the lungs. While it is clear that Y. pestis suppresses proinflammatory responses in general, it is not known precisely which of these host signals are necessary to suppress to establish an initial preinflammatory phase of disease. Similarly, while nearly any proinflammatory cytokine measured to date is highly induced during the later stages of infection, it is not clear precisely which host signals drive the transition to a proinflammatory disease stage. Here, we use an intranasal infection model to address this knowledge gap and identify host signaling molecules that contribute to neutrophil infiltration in the lungs and whose Pla-mediated suppression aids in limiting early host inflammatory responses. We show that the absence of Pla results in increased expression of genes encoding the proinflammatory cytokines IL-6 and IL-17 and the chemokine monocyte chemoattractant protein (MCP-1) early in the lungs during the preinflammatory phase of pneumonic plague. Using mice lacking expression of each of these molecules, we determined their respective contributions to the progression of pneumonic plague. We found that IL-17 contributes to neutrophil recruitment in the absence of Pla during the preinflammatory phase of infection, indicating that its suppression is critical to establishing an early preinflammatory phase of disease. Additionally, we found that IL-17 contributes to neutrophil infiltration during the later proinflammatory phase of infection. This positions IL-17 as a critical player in the progression of pneumonic plague, whose pattern of expression impacts host inflammatory responses and neutrophil infiltration in the lungs throughout the duration of infection.

## RESULTS

### Loss of Pla results in early expression of IL-6, IL-17A, and MCP-1.

**FIG 1:**
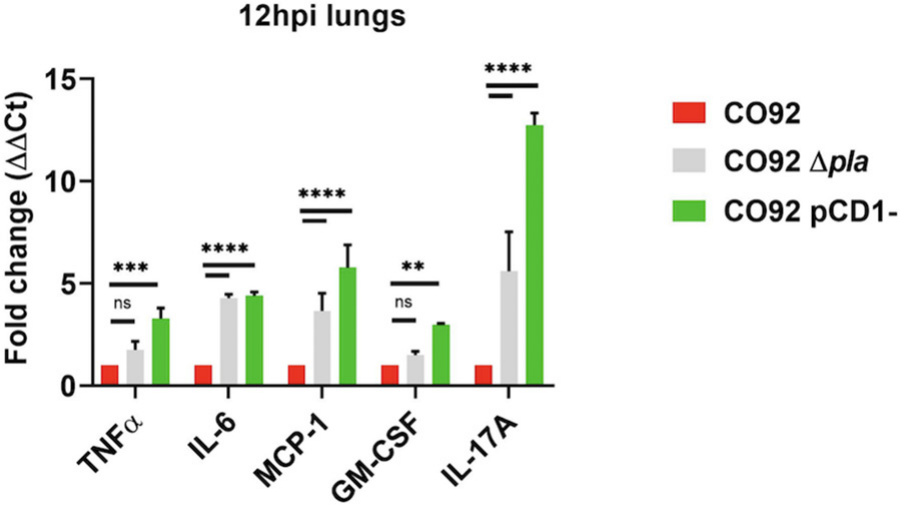
Regulation of proinflammatory cytokine expression in the absence of Pla. RNA isolated from whole lungs of C57BL/6J mice (*n* = 3) infected intranasally (i.n.) with 10^6^ CFU of WT CO92 Y. pestis, CO92 Y. pestis lacking pCD1^–^, or CO92 Δ*pla* 12 h postinfection (hpi) was used for qRT-PCR analysis of the relative expression of tumor necrosis factor alpha (TNF-α), IL-6, MCP-1, granulocyte-macrophage colony-stimulating factor (GM-CSF) and IL-17A during infection with pCD1^–^ or Δ*pla* strains relative to wild-type infection. Error bars represent standard deviations. The graph shows a representative experiment. **, *P ≤ *0.01; ***, *P ≤ *0.001; ****, *P ≤ *0.0001 (2-way analysis of variance).

Our lab showed that Pla suppresses neutrophil infiltration in the lungs early during infection through its function as an adhesin that facilitates T3S (13). While it is well established that the Y. pestis type 3 secretion system (T3SS) broadly suppresses innate immune responses largely through inhibition of NF-κB-mediated signaling (25, 26), it is less clear precisely which signals are necessary to suppress to establish a preinflammatory disease phase. The finding that deletion of Pla partially alleviates this early immune suppression afforded us the opportunity to identify those specific host molecules that are targeted to limit early neutrophil infiltration in the lungs. We predicted that measuring expression of proinflammatory cytokines and chemokines in the lungs of mice infected with Y. pestis lacking Pla (Δ*pla*) would reveal specific molecules that are otherwise suppressed during the earliest stages of wild-type infection (<24 h) that we could evaluate in greater detail. We analyzed expression of a number of host proinflammatory cytokines known to contribute to neutrophil chemotaxis via reverse transcription-quantitative PCR (qRT-PCR) during the early stages of infection with wild-type CO92 Y. pestis, Δ*pla*, or bacteria lacking the pCD1 virulence plasmid (27–30). Infection of mice with pCD1 Y. pestis occurs in the absence of T3S and therefore serves as a positive control for expression of target molecules. While these molecules had various levels of expression, we consistently observed a significant increase in IL-6, MCP-1, and IL-17 expression in the absence of Pla compared to wild-type infection (Fig. 1), suggesting that expression of these molecules is suppressed by Y. pestis to establish an early preinflammatory disease phase. We therefore sought to determine how expression of IL-6, MCP-1, and IL-17 impacted the biphasic progression of pneumonic plague.

### IL-17 drives early neutrophil infiltration into the lungs in the absence of Pla.

**FIG 2:**
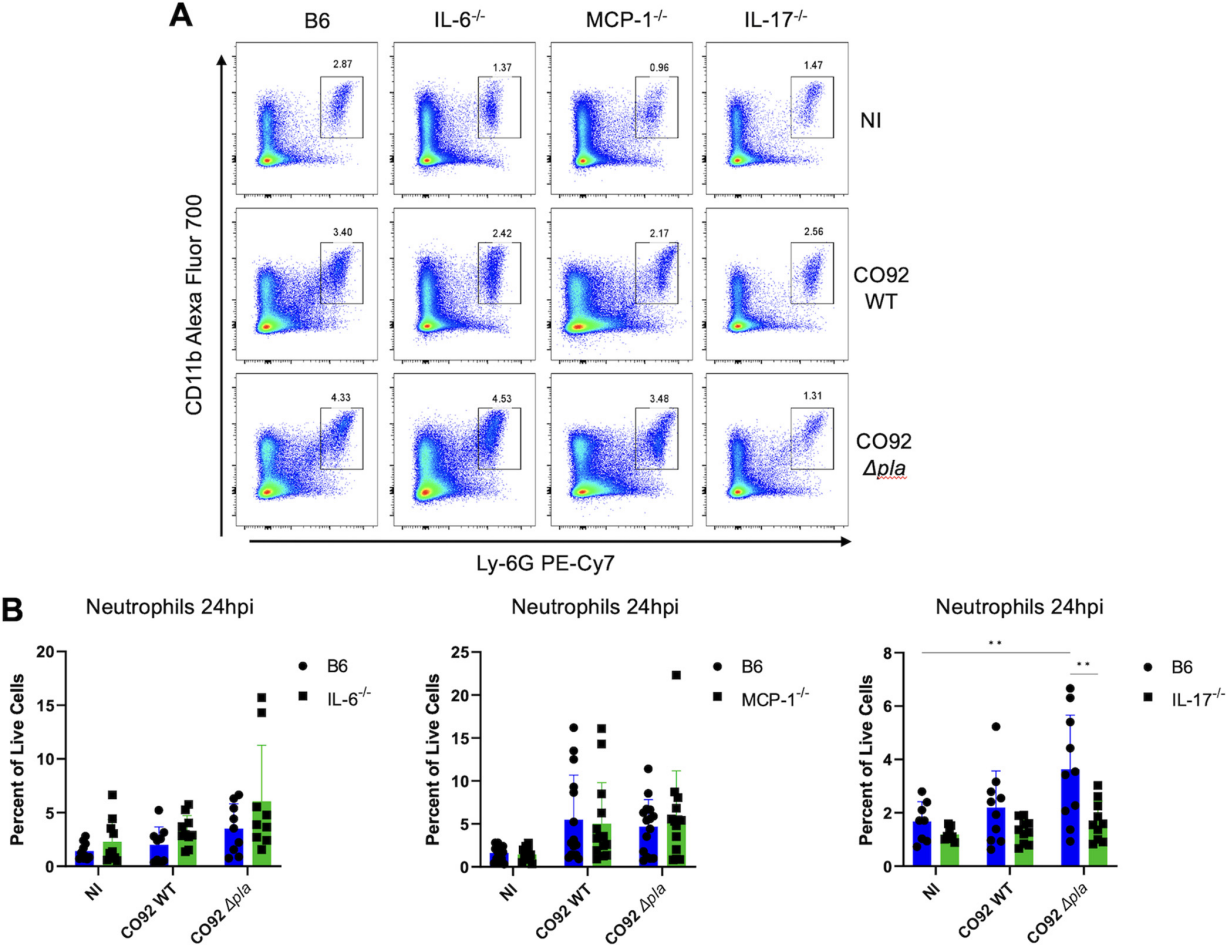
IL-17 is responsible for the influx of neutrophils during the preinflammatory phase seen in the absence of Pla. (A) F4/80^–^ neutrophil gate. Flow plots represent neutrophils as a percentage of live F4/80^–^ cells in the whole lung of IL-6, MCP-1, and IL-17 knockout mice or C57BL/6 WT mice infected i.n. with 10^4^ CFU of CO92 WT or CO92 Δ*pla* 24 hpi. (B) Quantification of the abundance of neutrophils (F4/80^–^CD11b^high^Ly-6G^+^) in the whole lungs of mice. The percentage of neutrophils out of all live cells is shown. Cy7, cyanine 7; PE, phycoerythrin. Experiments were performed ≥3 times, and graphs show the means from 2 to 3 pooled experiments; error bars represent the standard deviation (*n* = 10 to 15 mice). *, *P* ≤ 0.05; **, *P* ≤ 0.01 (2-way analysis of variance). The significance of the difference in lung neutrophil levels between IL-17 knockout mice and wild-type C57BL/6 mice infected with Δ*pla* Y. pestis was confirmed by Welch’s *t* test at *P* ≤ 0.05.

We recently showed that deletion of Pla results in the early migration of neutrophils into the lungs that is detected in the bronchoalveolar lavage fluid of infected mice as early as 12 h post infection (hpi) (13). This coincided with a significant increase in expression of IL-6, MCP-1, and IL-17 (Fig. 1). IL-6 and IL-17 are known to contribute to neutrophil chemotaxis during multiple infections/diseases (28, 31–34). IL-6 is promptly synthesized in infected tissue and contributes to neutrophil migration into tissue by modulating expression of chemokines, including MCP-1 and endothelial adhesion molecules (35) or by inducing neutrophil mobilization from the bone marrow (36, 37). Similarly, IL-17 has been shown to impact neutrophil recruitment, largely through its impact on chemokine expression and the release of neutrophil-mobilizing factors in local cells (38, 39). While it is known that MCP-1 contributes to migration and infiltration of monocytes/macrophages in a variety of diseases, its expression can also contribute to neutrophil chemotaxis during pulmonary infection (28). Previous work showed that pulmonary infection with a Y. pestis mutant strain that saw reduced neutrophil infiltration into the airways also saw reduced expression of four specific proinflammatory molecules (40). These included IL-6, MCP-1 and IL-17, further correlating expression of these molecules with neutrophil chemotaxis to the lung. We therefore hypothesized that the absence of one or more of these molecules would eliminate early aberrant neutrophil chemotaxis to the lung in the absence of Pla. To test this, we infected C57BL/6 mice lacking IL-6, MCP-1, or IL-17 (IL-6, MCP-1, or IL-17 knockout [KO] mice) intranasally (i.n.) with a lethal dose (10^4^ CFU) of wild type (WT) or Δ*pla* CO92 Y. pestis and evaluated levels of neutrophils in whole lungs by flow cytometry at 24 hpi. Analysis of whole lungs allows for simultaneous evaluation of host cell types, cytokines, and bacterial burdens. We observed no difference in lung neutrophil levels in IL-6 or MCP-1 KO compared to WT C57BL/6 mice during infection with either the WT CO92 or Δ*pla* strains (Fig. 2A and B). In contrast, the absence of IL-17 abrogated neutrophil migration to the lungs, and levels of lung neutrophils were significantly reduced in IL-17 KO mice during infection with Δ*pla* Y. pestis compared to WT mice (Fig. 2A and B). These results indicate that IL-17 contributes to neutrophil infiltration in the lung in the absence of Pla and suggests that the targeted suppression of IL-17 by Y. pestis aids in establishing an early preinflammatory disease phase.

### Absence of IL-6, MCP-1, or IL-17 did not impact growth of Δ*pla* Y. pestis in the lung.

**FIG 3:**
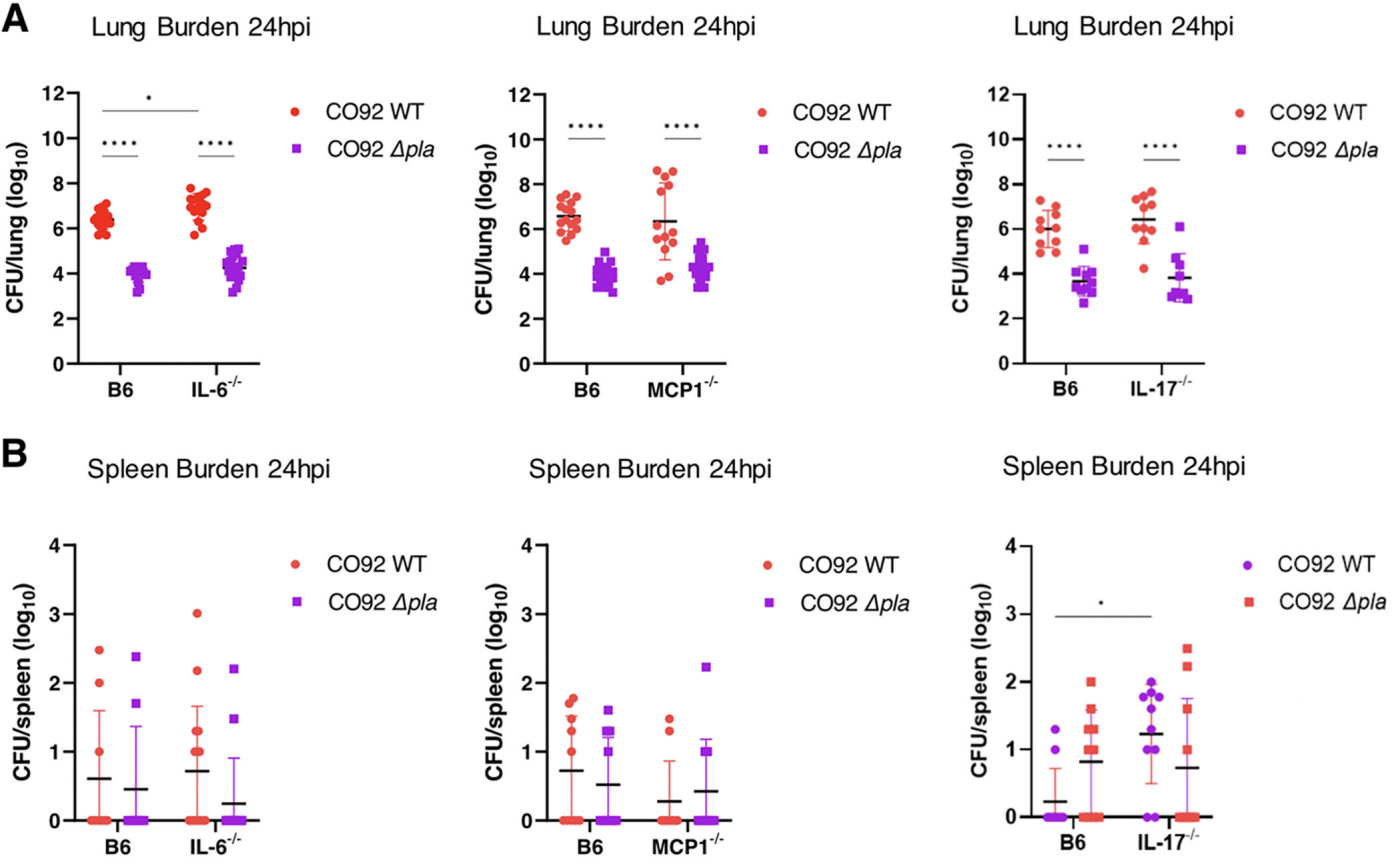
IL-17 inhibits bacterial dissemination early during CO92 WT infection. (A and B) Bacterial burdens in the lungs (A) or spleens (B) of IL-6, MCP-1, IL-17 knockout mice or WT C57BL/6 mice 24 h after i.n. infection with 10^4^ CFU of CO92 WT Yersinia pestis or Δ*pla* CO92. Experiments were performed ≥3 times, and graphs show 2 to 3 pooled experiments; error bars represent the standard deviation (*n* = 10 to 15 mice). *, *P* < 0.05; ****, *P ≤ *0.0001 (2-way analysis of variance).

Deletion of Pla results in decreased bacterial burdens in the lungs during i.n. infection of mice that is detectable as early as 12 hpi (12, 13). Depletion of neutrophils in mice prior to infection rescued growth of Δ*pla* Y. pestis at 24 hpi, indicating that the early influx of neutrophils likely controls Y. pestis in the absence of Pla (13). We sought to determine if the absence of either IL-6, MCP-1, or IL-17 alone could rescue growth and/or dissemination of Δ*pla* Y. pestis. We predicted that the absence of IL-17 would rescue Δ*pla* bacterial burdens due to reduced numbers of neutrophils in the lungs. As expected, we observed a significant decrease in lung bacterial burdens in WT B6 mice infected with Δ*pla* compared to WT Y. pestis at 24 hpi (Fig. 3A). The absence of IL-6, MCP-1, or IL-17 did not impact the growth of Δ*pla* Y. pestis relative to WT bacteria, indicating that neither of these cytokines alone drives responses capable of controlling infection (Fig. 3A). While the loss of MCP-1 and IL-17 had no effect on lung bacterial burden for either strain, the loss of IL-6 resulted in a slight but significant increase in the numbers of WT bacteria (Fig. 3A), indicating that IL-6 contributes to control of bacterial survival in the lung early during infection.

While there is minimal dissemination of bacteria beyond the lung during the preinflammatory disease phase (12), we also evaluated whether loss of a particular cytokine/chemokine of interest impacted dissemination of bacteria from the lung to other tissues as measured by the presence of bacterial burdens in the spleen. We observed no difference in dissemination in either CO92 WT or Δ*pla* Y. pestis in the absence of IL-6 or MCP-1 at 24 hpi (Fig. 3B). However, the loss of IL-17 resulted in increased dissemination of CO92 WT Y. pestis to the spleen, where 80% of mice harbored detectable bacterial burdens, suggesting a role for IL-17 in controlling bacterial dissemination from the lung during the preinflammatory phase (Fig. 3B). In summary, while the absence of IL-6, MCP-1, or IL-17 did not rescue growth of Δ*pla* Y. pestis, mice lacking IL-6 showed a slight increase in wild-type bacterial growth in the lung, and mice lacking IL-17 saw increased dissemination of wild-type bacteria to the spleen, indicating that these molecules contribute to bacterial control during pulmonary infection.

### Alveolar macrophages and neutrophils contribute to the production of IL-17 during the preinflammatory phase of disease.

**FIG 4:**
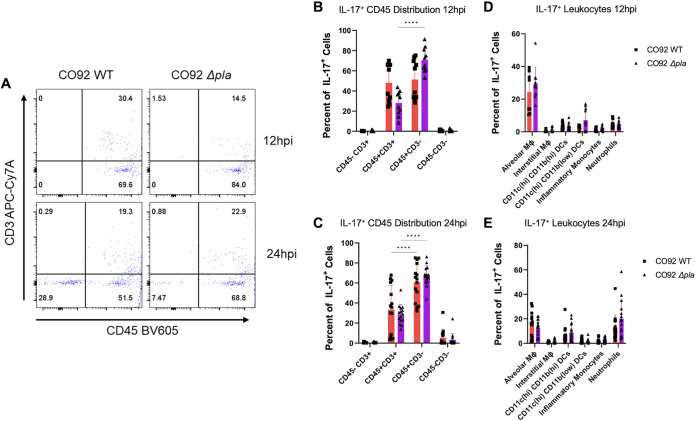
IL-17-producing cells during the preinflammatory phase of disease. (A) Flow cytometry plots representing CD45^+/–^/CD3^+/–^ populations as a percentage of live cells in the whole lung of C57BL/6 WT mice infected i.n. with 10^4^ CFU of WT or Δ*pla* CO92 at 12 or 24 h after infection. (B and C) Quantification of the abundance of IL-17^+^ CD45^+/–^/CD3^+/–^ populations in the whole lungs of mice as described in panel A. Graph represents the population as a percentage of IL-17^+^ cells. (D and E) Quantification of the abundance of IL-17^+^ CD45^+^CD3^–^ leukocytes in the whole lung of mice as described in panel A. Plot represents the population as a percentage of IL-17^+^ cells. Populations were identified as alveolar macrophages (F4/80^+^CD11c^high^CD11b^low^), interstitial macrophages (F4/80^+^CD11c^low^CD11b^high^), dendritic cells (F4/80^–^CD11c^high^CD11b^high or low^), monocytes (F4/80^–^CD11b^high^CD11c^low^Ly-6G^–^), and neutrophils (F4/80^–^CD11b^high^Ly-6G). APC, allophycocyanin; Cy7, cyanin 7; BV, brilliant violet. All experiments were performed ≥3 times, and graphs show 2 pooled experiments. Error bars represent standard deviations (*n* = 10). ****, *P ≤ *0.0001 (2-way analysis of variance).

To better understand the expression of IL-17, we used flow cytometry coupled with intracellular cytokine staining to identify the cell types responsible for IL-17 production during the preinflammatory phase of infection. Previous work utilizing two strains of the Microtus biovar of Y. pestis showed increased expression of IL-17 produced by CD45^+^ cells between 12 and 24 hpi and determined that neutrophils are a major source of IL-17 during infection (24). At 12 hpi, IL-17 production was split equally between CD45^+^CD3^+^ and CD45^+^CD3^–^ populations during WT infection (Fig. 4A and B). CD45^+^CD3^+^ cells include lymphocyte Th17 and natural killer T cell populations that are well-established producers of IL-17 (41). CD45^+^CD3^–^ populations include resident lung leukocytes such as alveolar macrophages, interstitial macrophages, and dendritic cells, as well as neutrophils and inflammatory monocytes. Interestingly, at 12 hpi with Δ*pla* Y. pestis there was a shift to a significantly higher percentage of IL-17 expression for CD45^+^CD3^–^ cells compared to CD45^+^CD3^+^ populations (Fig. 4A and B). Using a fluorescent antibody panel to identify CD45^+^CD3^–^ leukocyte populations in the lung, we found that alveolar macrophages were significant producers of IL-17 at 12 hpi during both WT and Δ*pla* infection (Fig. 4D). At 24 hpi CD45^+^CD3^+^ and CD45^+^CD3^–^ remained the primary producers of IL-17 (Fig. 4C), and both Δ*pla* and WT infection saw CD45^+^CD3^–^ cells producing a significantly higher percentage of IL-17 than that in CD45^+^CD3^+^ populations. We found that in addition to alveolar macrophages, neutrophils were also significant producers of IL-17 at 24 hpi (Fig. 4E). This coincides with the migration of neutrophils to the alveolar space that is observed at 12 to 24 hpi (13). Of note, at 24 hpi we began to observe IL-17 production from a population of CD45-CD3^–^ cells, indicating that endothelial/epithelial cells may play a role in IL-17 signaling during the mid/later stages of the preinflammatory disease phase. We also observed IL-17 expression from dendritic cell (DC) populations at both time points, highlighting the importance of the initial signaling by lung antigen-presenting cells during the earliest stages of infection. These results suggest that early during infection alveolar macrophages produce IL-17 to recruit an initial wave of neutrophils that also produce IL-17.

### IL-17 contributes to neutrophil recruitment during the proinflammatory phase of infection.

**FIG 5:**
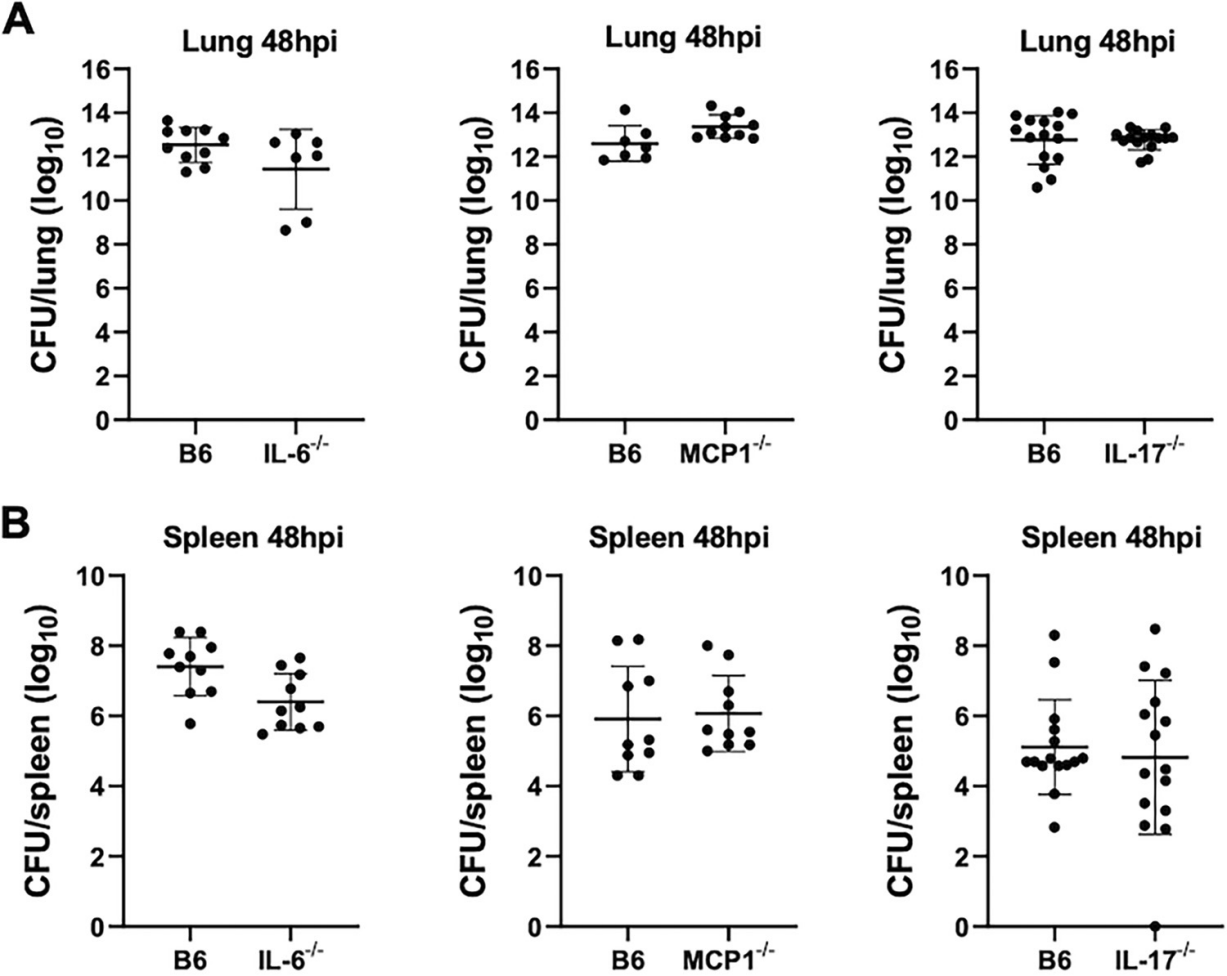
IL-17 does not impact bacterial burden in the lung or dissemination to the spleen during the proinflammatory phase of disease. (A and B) Bacterial burdens in the (A) lungs and (B) spleen of IL-6, MCP-1, and IL-17 knockout mice or C57BL/6 mice 24 h after i.n. infection with 10^4^ CFU of WT CO92 Yersinia pestis. All experiments were performed ≥3 times, and graphs show 2 to 3 pooled experiments. Error bars represent standard deviations (*n* = 10 to 15). *, *P* < 0.05 (Welch’s *t* test).

**FIG 6:**
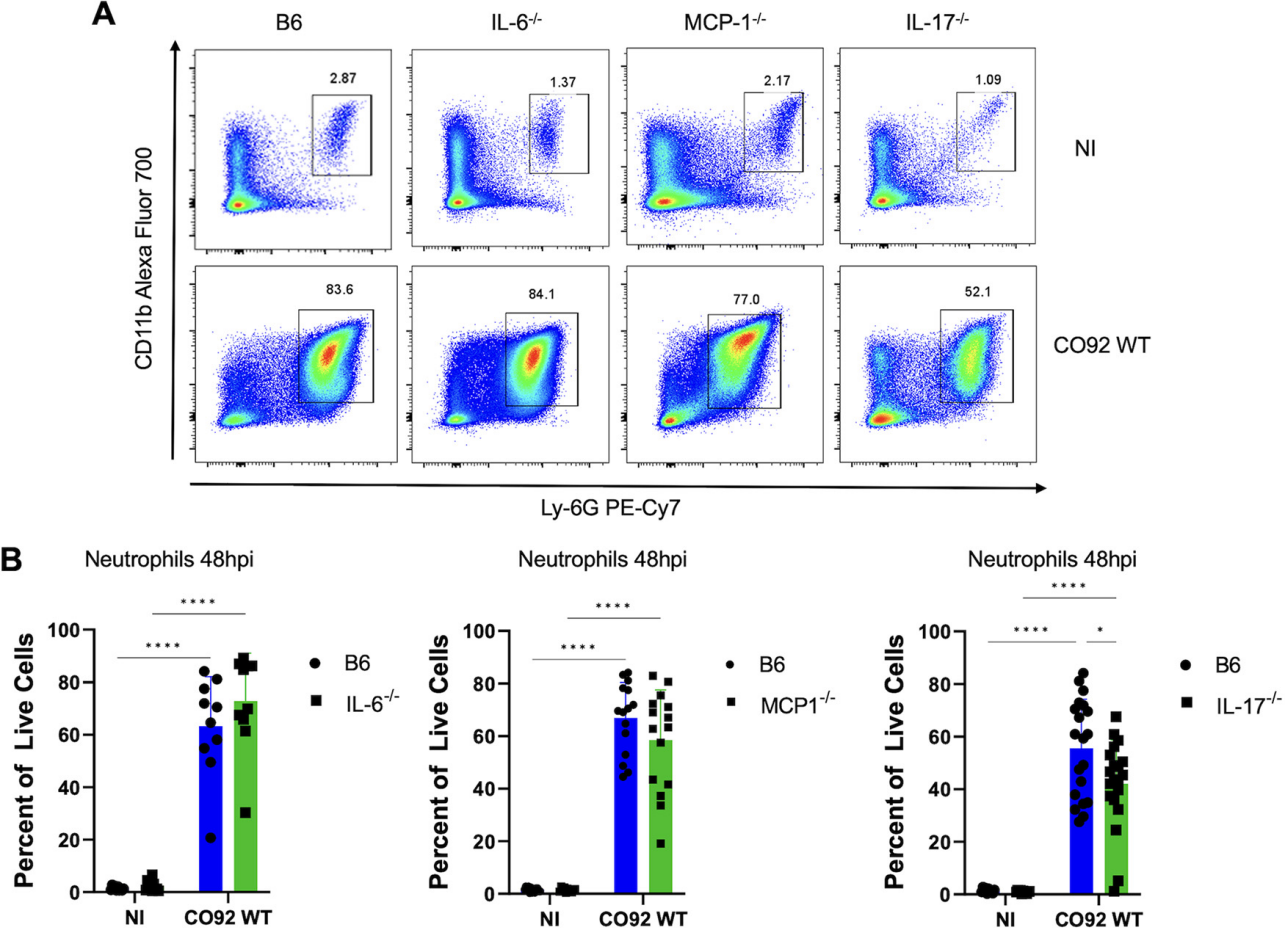
IL-17 contributes to neutrophil recruitment during the proinflammatory phase of disease. (A) F4/80^–^ neutrophil gate. Flow cytometry plots represent neutrophils as a percentage of live F4/80^–^ cells in the whole lung of IL-6, MCP-1, and IL-17 knockout mice or C57BL/6 mice 48 h after intranasal infection with 10^4^ CFU of CO92 WT Y. pestis. (B) Quantification of the abundance of neutrophils (F4/80^–^CD11b^high^Ly-6G^+^) in the whole lungs of mice. The percentage of neutrophils out of all live cells is shown. Cy7, cyanine 7; PE, phycoerythrin. All experiments were performed ≥3 times, and graphs show the means from 2 to 3 pooled experiments. Error bars represent standard deviations (*n* = 10 to 15). *, *P* < 0.05; ****, *P ≤ *0.0001 (2-way analysis of variance). The significance of the difference in lung neutrophil levels between IL-17 knockout mice and wild-type C57BL/6 mice was confirmed by Welch’s *t* test at *P* ≤ 0.05.

**FIG 7:**
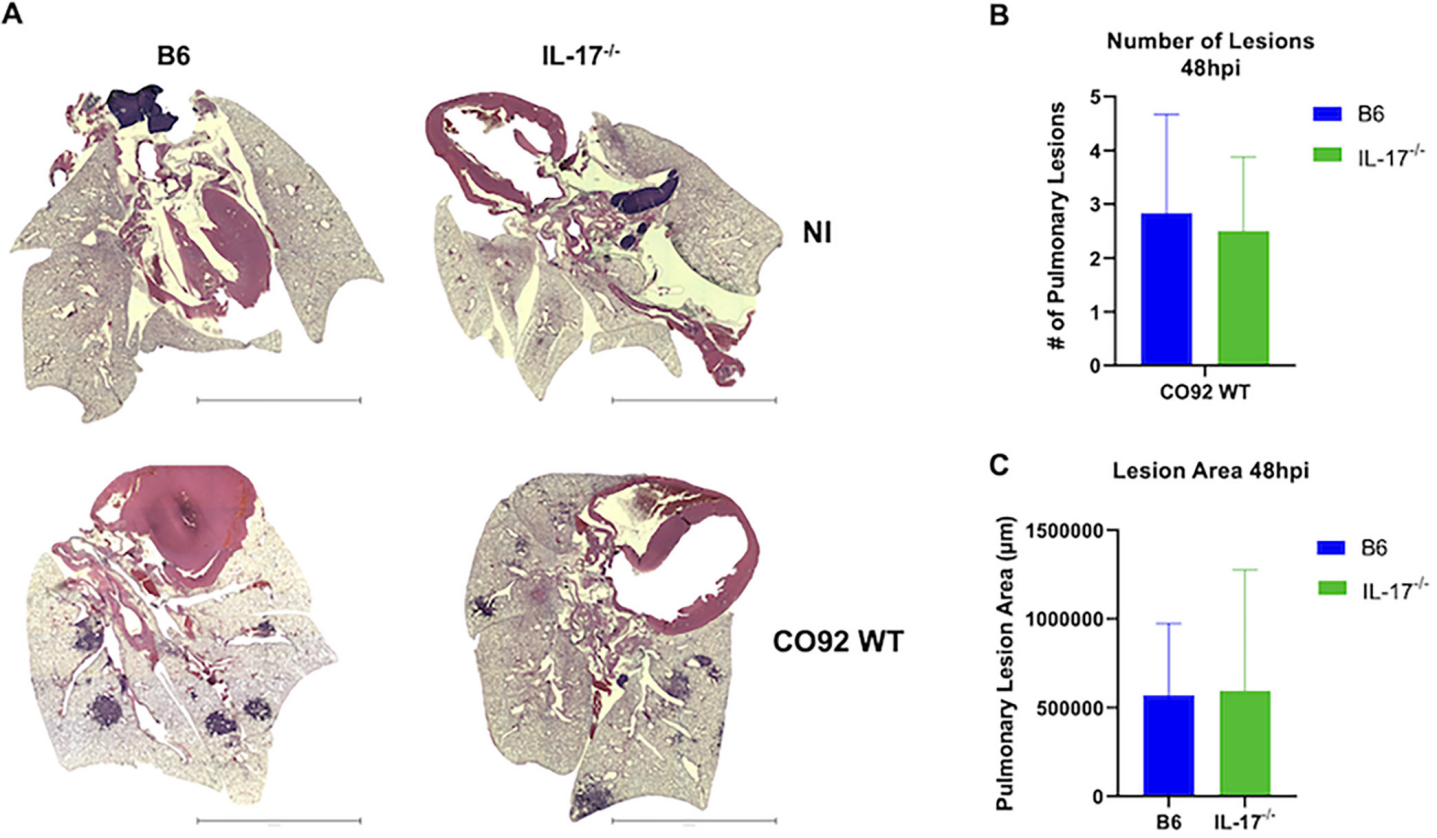
Loss of IL-17 does not affect pulmonary lesion formation or size during the proinflammatory disease phase. (A) Representative hematoxylin and eosin (H&E) images of whole lungs of uninfected mice or mice infected with 10^4^ CFU of CO92 WT 48 hpi. Images show a lung from a single representative mouse per group. Scale bar = 3,000 μm. (B) Number of pulmonary lesions based on H&E staining of lungs from B6 and IL-17^−/−^ mice infected with 10^4^ CFU CO92 WT Y. pestis. (C) Lesion size (μm) was determined using ImageJ. All experiments were performed 2 times. Error bars represent standard deviations (*n* = 6). *, *P* < 0.05 (Welch’s *t* test).

The proinflammatory disease phase of pneumonic plague is marked by a dramatic influx of neutrophils into the lungs accompanied by the onset of a proinflammatory cytokine storm (3, 8, 42). It remains unclear, though, precisely which host signals drive neutrophil infiltration and progression into the proinflammatory phase of disease. In addition to early events in the lung during Δ*pla* infection, expression of IL-6, IL-17, and MCP-1 has been previously shown to correlate with levels of pulmonary neutrophils during the later proinflammatory stage of infection (40). We therefore sought to examine the role of IL-6, IL-17, and MCP-1 in the transition to a proinflammatory state in the lung during infection. The Δ*pla* strain has attenuated growth in the lung beginning at 12 hpi and does not progress to the proinflammatory stage of disease; therefore, WT infection alone was used to evaluate the later proinflammatory stage of infection. Mice lacking IL-6, IL-17, or MCP-1 showed no difference in lung bacterial burden compared to WT mice infected with Y. pestis, nor was there a difference in dissemination to the spleen, suggesting that IL-17 does not contribute to control of bacterial survival or dissemination during the proinflammatory disease phase (Fig. 5A and B). However, the absence of IL-17 saw significantly decreased levels of neutrophils in the lungs compared to WT mice (Fig. 6A and B). Despite a roughly 20% decrease in lung neutrophil levels, the absence of IL-17 did not appear to impact the presence and/or organization of the neutrophil-packed inflammatory lesions that are a hallmark of pneumonic plague (Fig. 7A) (4). While inflammatory lesions appeared to be less densely packed and borders were more poorly demarcated, we did not detect a difference in the number or size of pulmonary lesions between WT mice and IL-17^−/−^ mice (Fig. 7B and C). These results suggest that IL-17 contributes to the recruitment of neutrophils during the proinflammatory disease phase, but IL-17 alone is not responsible for the inflammatory pathology seen during the later stages of disease.

## DISCUSSION

The protease Pla functions as an adhesin to facilitate T3S of the Yop effectors into host alveolar macrophages to silence host innate immune responses (11, 13, 14, 18). The absence of Pla results in increased neutrophil influx into the lung early during infection (13). This positioned Pla as an important factor in maintaining the preinflammatory phase of Y. pestis infection. While Y. pestis is efficient at silencing host inflammatory responses overall, the specific molecules that need to be suppressed to limit early neutrophil infiltration into the lungs remain unknown and therefore represent a knowledge gap in the field. In this study, we used Y. pestis lacking Pla as a tool to identify host factors that are typically “silenced” in the lung by Pla-mediated T3S during the earliest stages of infection. Using an i.n. murine infection model of pneumonic plague, we found that partially alleviating early suppression of host responses by deleting Pla resulted in increased expression of host IL-6, MCP-1, and IL-17 during the early preinflammatory phase of disease. We coupled infection using knockout mice and a bacterial mutant strain to characterize the role of each of these molecules in the host responses that define the biphasic progression of pneumonic plague. While the absence of IL-6 and MCP-1 did not significantly alter the progression of disease, we identified IL-17 as a key cytokine that impacts host inflammatory responses during the progression of primary pneumonic plague.

IL-17 is a proinflammatory cytokine that has neutrophil chemotactic properties and has previously been shown to be highly expressed during the proinflammatory phase of pneumonic plague (13, 29, 30, 41). IL-17 has also been shown to be an important mediator of protection in vaccination studies of pneumonic plague, typically in synergy with other molecules such as gamma interferon (IFN-γ) (43, 44). Our data show that IL-17 is suppressed by Pla-mediated T3S early during infection, contributing to inhibition of neutrophil migration to the lungs. A previous study by Bi et al. showed that IL-17 KO mice have increased lung bacterial burdens compared to WT mice during pneumonic plague (24). However, we did not find that IL-17 impacted bacterial survival. A likely explanation for this is that Bi et al. utilized strains from a different biovar of Y. pestis with a different lethal dose and different growth kinetics during infection (24). Bi et al. used strains of the biovar Microtus that saw peak bacterial burdens of roughly 10^8^ CFU at 4 days postinfection in the lungs of infected mice and nearly 100% survival using similar inoculating doses as our study (24). In this scenario, the investigators found that IL-17 was protective and prevented lethality. In contrast, we used CO92 of the Orientalis biovar, infection with which results in 100% lethality by 3 days postinfection (8, 12, 45) and >10^12^ CFU in the lungs at 48 hpi. In our study IL-17 is not protective and, alternatively, may contribute to disease severity later during infection through its contribution to neutrophil accumulation in the lungs. These data suggest that IL-17 may play different roles depending on the *Yersinia* biovar or relative lethality of the infecting bacterial strains. For less lethal biovars such as Microtus, IL-17-driven neutrophil chemotaxis may be protective, while during infection with Orientalis strains neutrophil infiltration likely contributes to the severity of pulmonary disease and therefore lethality. Further work will need to be done to further characterize this phenomenon, but it highlights the fact that specific host responses can be protective or exacerbate disease depending on the lethality/severity of the infecting strain or the susceptibility of the host.

Previous data from our lab showed that the increased neutrophil infiltration in the lungs upon deletion of Pla limited replication of the Pla mutant during the preinflammatory stage of infection (13). Antibody-mediated depletion of neutrophils prior to infection rescued growth of Δ*pla* Y. pestis by 24 hpi, and we showed that Pla contributes to bacterial resistance to neutrophil-mediated killing. However, in this study decreased neutrophils in the lung due to the loss of IL-17 did not impact the growth of CO92 Δ*pla*. The significance of this finding is not yet clear. Perhaps neutrophil populations other than those recruited by IL-17 (which are therefore still present in IL-17 KO mice) are responsible for limiting the growth of Δ*pla* Y. pestis. Bi et al. showed that IL-17 signaling contributes to M1 macrophage programming that is protective during pneumonic plague in mice (24). The Y. pestis CO92 strain, though, may require a combination of additional mechanisms/signals to limit bacterial growth. Though significant, the increase in neutrophil infiltration during infection with Δ*pla* is subtle, especially compared to the dramatic influx of innate immune populations later during infection. It may be that this increase and the subsequent decrease in the absence of IL-17 are not sufficient to impact bacterial growth and/or survival. Regardless, the precise mechanism responsible for controlling Δ*pla* Y. pestis remains unclear and is likely multifactorial. The absence of IL-17 did, though, result in enhanced WT bacterial burdens in the spleen, indicating that IL-17 contributes to controlling bacterial dissemination from the lung to other tissues. Also, loss of IL-6 resulted in a slight but significant increase in lung bacterial burden during wild-type Y. pestis infection. Current work is focused on characterizing the impact of IL-17 on downstream innate immune signaling during the early stages of pneumonic plague and the combination of host signals that need to be suppressed by Y. pestis to establish a replicative niche in the lung.

IL-17 has been described as being secreted by various cell types in the lung, including Th17, CD8^+^ T cells, innate immune cells, etc.; however, the source of IL-17 during infection varies from pathogen to pathogen (19–23). Our study examined the sources of IL-17 during infection with Y. pestis and found that the cells secreting IL-17 during the initial stages of infection include a significant contribution from alveolar macrophages. This aligns with previous data from our lab and suggests that reduced targeting of alveolar macrophages for T3S likely results in IL-17 secretion (14). Alveolar macrophages are resident to the lung (46) and one of the first cells to make contact with the bacteria (8), thus initiating the production of IL-17 to recruit the first wave of neutrophils, which in turn, assume a role in secreting IL-17. This aligns with the data presented by Yujing Bi and colleagues, who described neutrophils as secreting IL-17 during Y. pestis infection (24). We also observed IL-17 secretion for DCs, potentially highlighting a yet to be described role for these cell populations in host responses that define the progression of pneumonic plague. While our study only expanded on the CD45^+^CD3^–^ leukocyte populations that produce IL-17, it is of note that there are additional populations that contribute to IL-17 expression that have yet to be characterized in detail during pneumonic plague. While WT infection saw equal contributions from CD45^+^CD3^+^ and CD45^+^CD3^–^ populations in IL-17 production at 12 hpi, the absence of Pla saw a significant shift in that balance, with CD45^+^CD3^–^ populations producing increased IL-17 compared to CD45^+^CD3^+^ populations. Interestingly, at 24 hpi we observed a contribution from CD45^–^CD3^–^ cells in IL-17 production, which includes endothelial/epithelial populations. This gives insight into the temporal production of IL-17, where the earliest time point sees resident alveolar macrophages as the primary producer, and a shift to include neutrophils and, potentially, CD45^–^CD3^–^ endothelial/epithelial cells beginning 12 h later. Future studies will be performed to gain a clear picture of the players and timing involved in the secretion of IL-17 during the critical preinflammatory stage of pneumonic plague.

A hallmark of primary pneumonic plague is the progression into a lethal proinflammatory phase of infection (3–7). The proinflammatory phase occurs around 36 h in mice and is marked by uncontrolled proinflammatory cytokine production leading to a rapid influx of neutrophils, damage to the alveolar architecture, and diminished pulmonary function and ultimately resulting in death (3–7). The onset of a proinflammatory cytokine storm during pneumonic plague is well established, and nearly any proinflammatory cytokine or chemokine measured during this stage of infection shows dramatically increased expression (3, 4, 7, 12, 40). It is not known, though, precisely which of these molecules/signals is responsible for the dramatic neutrophil chemotaxis to the lungs that defines the proinflammatory stage of infection. IL-17 has been described as one of the cytokines most highly upregulated during the proinflammatory disease phase (12, 24), and we discovered that while it does not control bacterial survival in the lung, it does contribute to the massive influx of neutrophils during the proinflammatory disease phase (1, 3, 8, 12). This implicates IL-17 as a player in the transition to a lethal proinflammatory state of infection in the lung and has therapeutic implications, as limiting pulmonary inflammation (including neutrophil infiltration) has been shown to enhance treatment of late-stage infection (47). However, the loss of IL-17 did not impact the formation and organization of inflammatory pulmonary lesions, not surprisingly indicating that progression into the proinflammatory disease phase is multifactorial and involves expression of IL-17 in tandem with other signals.

This work has shown that alveolar macrophage- and neutrophil-derived IL-17 is suppressed by Pla-mediated T3S early during infection to establish an initial preinflammatory stage of infection. Further, the upregulation, or rather, “derepression” of IL-17 expression contributes to neutrophil infiltration in the lungs and progression to a lethal proinflammatory stage of infection. While previous work had defined a protective role for neutrophil-derived IL-17 during infection with *Yersinia* of the Microtus biovar, we did not find that IL-17 controls bacterial growth and/or survival, and it is likely not protective during infection with the highly lethal CO92 strain of the Orientalis biovar. In summary, this work indicates that IL-17 is a critical cytokine, the expression of which contributes to the biphasic progression of pneumonic plague. Targeting IL-17 in tandem with antibiotic therapy may aid in treating late-stage pneumonic plague or pneumonia caused by other agents in which IL-17-driven neutrophil infiltration in the airways contributes to pathogenesis.

## MATERIALS AND METHODS

### Bacterial strains.

Y. pestis fully virulent strain CO92 (biovar Orientalis), pCD1- CO92, and Δ*pla* CO92 (12) were obtained from William E. Goldman (University of North Carolina at Chapel Hill). All experiments were performed in a biosafety level 3 facility at the University of Arkansas for Medical Sciences. Y. pestis strains were grown on brain heart infusion (BHI) agar (Difco) at 26°C for 2 days. For infection, Y. pestis strains were grown in 10 mL of BHI broth containing 2.5 mM calcium chloride for 16 h at 37°C with constant shaking.

### Animal infections.

All animal experiments were conducted as approved by the University of Arkansas for Medical Sciences (UAMS) Institutional Animal Care and Use Committee. Female C57BL/6, IL-6 knockout (B6.129S2-*Il6^tm1Kopf^/*J), MCP-1 knockout (B6.129S4-*Ccl2^tm1Rol^/*J), and IL-17 knockout (B6.129 (SJL)-*Il17a^tm1.1 (icre)Stck^/*J) mice (4 to 6 weeks old) were obtained from Jackson Laboratories. Mice were given food and water and maintained at 25 to 26°C with 40 to 70% humidity. For infection, mice were anesthetized via the intraperitoneal (i.p.) route using ketamine-xylazine. Anesthetized mice were inoculated via the intranasal (i.n.) route with a lethal dose (10^4^ to 10^6^ CFU) of Y. pestis delivered in 20 μL phosphate-buffered saline (PBS). Mice were sacrificed using a lethal dose of sodium pentobarbital injected i.p. Bacterial burdens were determined via harvesting and homogenizing the lungs and spleens of mice at 24 h and 48 h postinfection. Organ homogenates were serially diluted and plated on BHI agar to determine CFU.

### Flow cytometry.

Mouse lungs were digested in collagenase solution (1.5 mg/mL collagenase type IV, 0.4 mg/mL DNase1, 10 mM HEPES, and 5% fetal bovine serum [FBS] in Hanks’ balanced salt solution) for 1 h. Tissue was mechanically sheared using dissection scissors, and the suspension was passed through a 70-μM mesh filter. Cells were centrifuged at 500 × *g* for 5 min, washed once, and resuspended in 1× red blood cell (RBC) lysis buffer for 5 min. The suspension was diluted to 10 mL with PBS before being centrifuged again. Cells were resuspended in 1 mL of Live/Dead fixable aqua dead cell stain diluted in 1× PBS for 30 min at room temperature before being stained in PBS with 3% FBS (3% FBS/PBS) containing the following cell surface markers (1:500 dilution) for 30 min at 4°C: CD45-phycoerthrin (clone 30-F-11, BD Biosciences), CD3-allophycocyanin-Cy7 (clone 17A2, BD Biosciences), CD11b-Alexa Fluor 700 (clone M1/70, BD Biosciences) CD11c-brilliant violet 786 (clone HL3, BD Biosciences), F4/80-allophycocyanin (clone BM8, Invitrogen), and Ly6G-phycoerthrin-Cy7 (clone 1A8, BD Biosciences). Following staining, cells were centrifuged as previously described and fixed in 300 μL of 2% formalin in PBS for 15 min at room temperature prior to removal from the biosafety level 3 (BSL3) facility. Stained cells were analyzed based on fluorescence staining patterns to identify alveolar macrophages (F4/80^+^CD11b^mid/low^CD11c^high^), CD11b^high^ interstitial/exudate macrophages (F4/80^+^CD11b^high^CD11c^low/mid^), monocytes (F4/80-CD11b^high^CD11c^low^Ly-6G^–^), CD11b^high^ and CD11b^low^ dendritic cells (F4/80^–^CD11c^high^CD11b^high or low^), and neutrophils (F4/80^–^CD11c^low^CD11b^high^Ly-6G^+^) (8). The general gating strategy to identify different cell types is highlighted in Fig. S1 in the supplemental material. For intracellular cytokine staining, prior to surface staining processed lungs were incubated with brefeldin A solution for 3 h at 37°C with 5% CO_2_, treated with BD Cytofix/Cytoperm buffer for 20 min at 4°C, and stained with antibodies specific for IL-17A (clone TC11-18H10.1, BioLegend) in permeabilization (perm)/wash buffer for 60 min at 4°C before resuspension in 300 μL 3% FBS/PBS. Data were analyzed with FlowJo software v10.8.1.

### Lung pathology.

After sacrifice, mouse lungs were inflated with 1 mL 10% formalin via cannulation of the trachea and harvested at 48 h postinfection (hpi) (48). Lungs were placed in 10 mL 10% formalin and submitted to the UAMS Experimental Pathology Core for paraffin embedding, tissue slicing and mounting, and hematoxylin and eosin staining. Three sections per lung (each section cut 100 μm apart) were obtained. Lung lesions were quantified using microscopy. An Evos FL Auto 2 instrument was used to scan slides, and stitched images were analyzed using ImageJ (NIH).

### Quantitative reverse-transcription PCR.

Groups of mice were inoculated i.n. with 10^6^ CFU of CO92 WT, CO92 pCD1-^–^, or CO92 Δ*pla* Y. pestis, and at 12 hpi lungs were harvested, placed in 1 mL of PBS, and homogenized using a tissue tearer. Homogenized lungs were centrifuged at 500 × *g* for 5 min, supernatant was removed, and the pellet was lysed in TRIzol reagent (Thermo Fisher). Total RNA was then isolated from lung homogenates per the manufacturer’s instructions and treated with DNase I using the TURBO DNA-*free* kit (Invitrogen). Then, 1 μg of RNA was reverse transcribed to generate cDNA using the Superscript III polymerase kit (Invitrogen). Real-time quantitative reverse transcription PCR was performed using PowerUp SYBR green master mix (Thermo Fisher) with a QuantStudio 6 system, and fold changes were calculated using the delta delta cycle threshold method normalized to glyceraldehyde-3-phosphate dehydrogenase (GAPDH) from the same sample.

### Statistical analysis.

All statistical analyses were done using 2-way analysis of variance or Welch’s *t* test. All statistical analyses were performed using GraphPad Prism software v9.4.
