# Supplementary material for: Pulmonary Expression of Interleukin-17 Contributes to Neutrophil Infiltration into the Lungs during Pneumonic Plague
Source: Infect Immun. 2023 Jun 20;91(7):e00131-23. doi: 10.1128/iai.00131-23 (PMC10353359; doi:10.1128/iai.00131-23)
Supplement: Supplemental file 1 — Fig. S1. Download iai.00131-23-s0001.pdf, PDF file, 1.6 MB [file iai.00131-23-s0001.pdf]

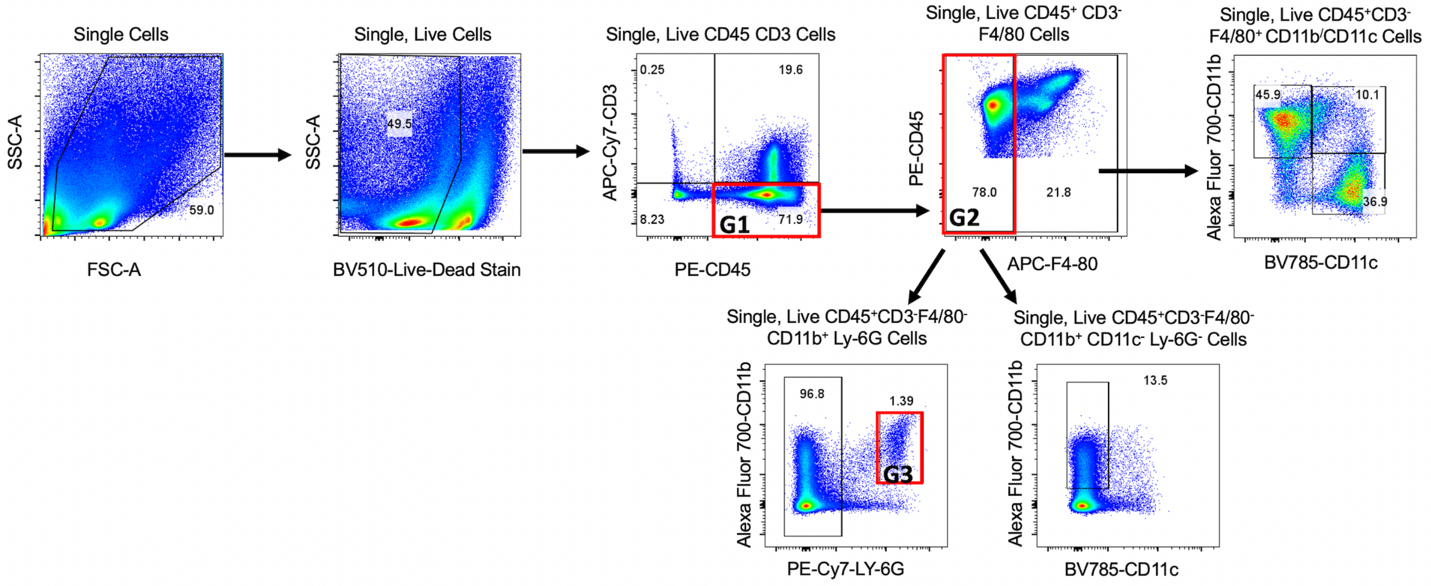

Figure S1. Gating Scheme for Flow Cytometry.

The gating strategy used to identify various host cell types is shown in representative histograms from a mouse infected with wild type CO92 *Y. pestis*. G1=live $CD3^{-}CD45^{+}$ cells, G2= live $CD3^{-}CD45^{+}F4/80^{-}$ cells, G3= $CD3^{-}CD45^{+}F4/80^{-}$Ly-6G+ neutrophils. A plot for CD11b and CD11c is shown for both $F4/80^{-}$ (DCs) and $F4/80^{+}$ (macrophage) populations.
